# Supplementary material for: DMRforPairs: identifying Differentially Methylated Regions between unique samples using array based methylation profiles
Source: BMC Bioinformatics. 2014 May 15;15:141. doi: 10.1186/1471-2105-15-141 (PMC4046028; doi:10.1186/1471-2105-15-141)
Supplement: Additional file 2 — DMRforPairs output for the comparison of A431-MCF7 and NA17018-NA17105. Please start from the HTML files in each folder. Available via the BMC Bioinformatics website. [file 1471-2105-15-141-S2.zip › 1394847754114233_MOESM2_ESM/A431_MCF7/figures/19203.pdf]

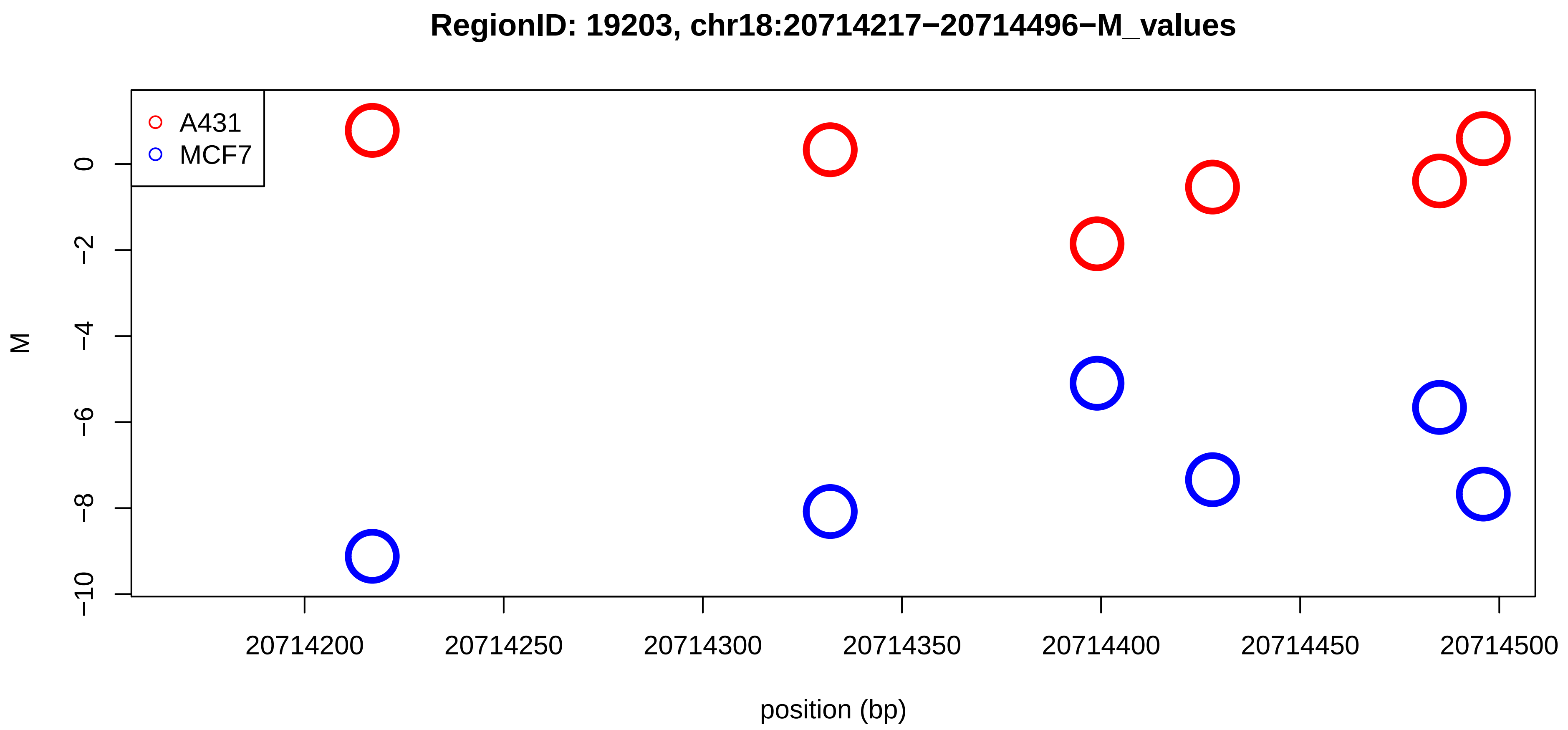

RegionID: 19203, chr18:20714217−20714496−M_values
A431
MCF7
M
0
−2
−4
−6
−8
−10
20714200
20714250
20714300
20714350
20714400
20714450
20714500
position (bp)

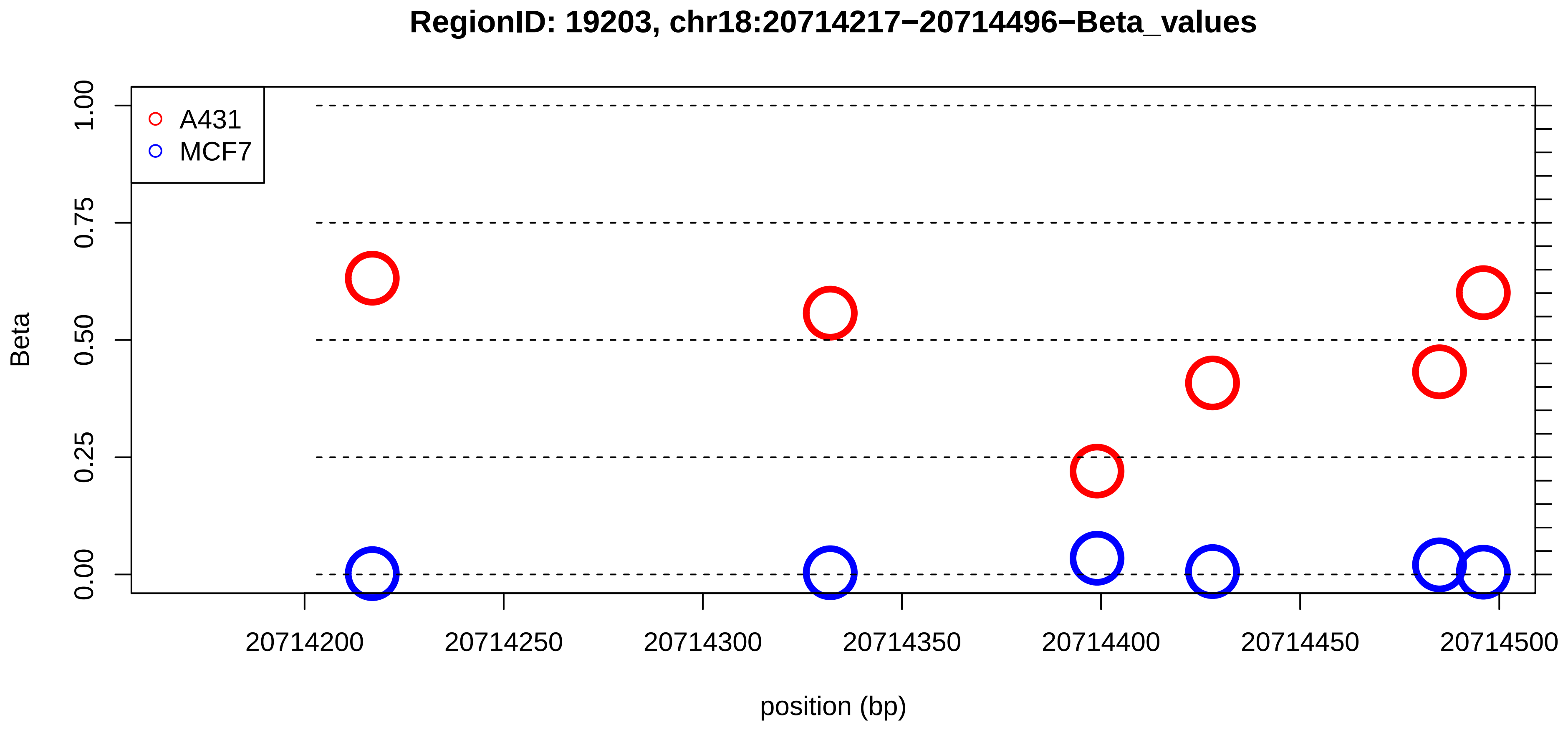

RegionID: 19203, chr18:20714217−20714496−Beta_values
A431
MCF7
Beta
1.00
0.75
0.50
0.25
0.00
20714200
20714250
20714300
20714350
20714400
20714450
20714500
position (bp)

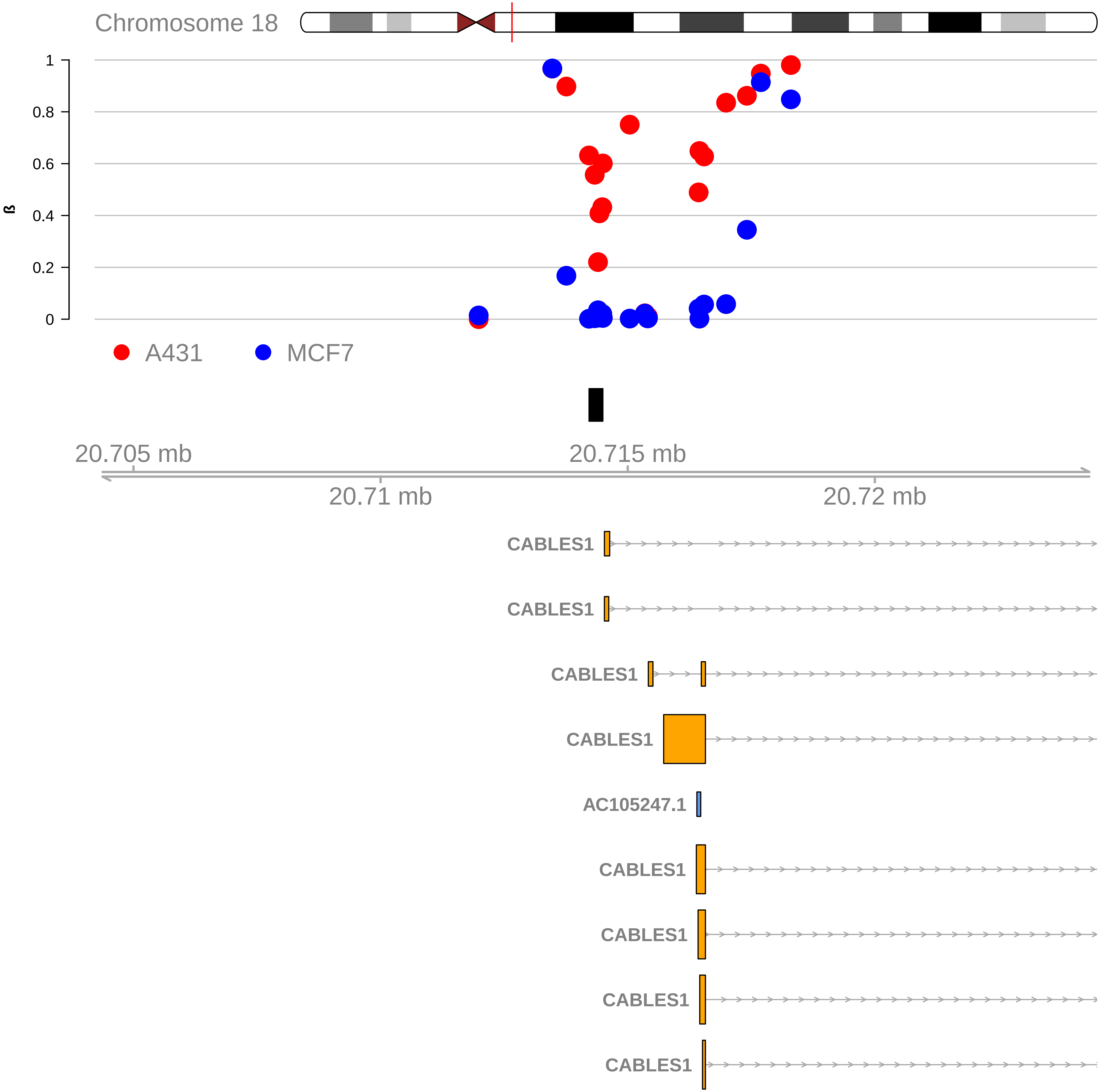

Chromosome 18
1
0.8
0.6
0.4
0.2
0
β
A431
MCF7
20.705 mb
20.71 mb
20.715 mb
20.72 mb
CABLES1
CABLES1
CABLES1
CABLES1
AC105247.1
CABLES1
CABLES1
CABLES1
CABLES1
